# Two-stage analysis strategy for identifying the IgM quantitative trait locus

**DOI:** 10.1186/1753-6561-1-s1-s139

**Published:** 2007-12-18

**Authors:** Tao Wang, Qing Lu, Monica Torres-Caban, Robert C Elston

**Affiliations:** 1 Department of Epidemiology and Biostatistics, Case Western Reserve University, 2103 Cornell Road, Cleveland, Ohio 44106, USA

## Abstract

Genetic association studies offer an opportunity to find genetic variants underlying complex human diseases. Various tests have been developed to improve their power. However, none of these tests is uniformly best and it is usually unclear at the outset what test is best for a specific dataset. For example, Hotelling's *T*^2 ^test is best for normally distributed data, but it can lose considerable power when normality is not met. To achieve satisfactory power in most cases, without compromising the overall significance level, we propose to adopt a two-stage adaptive analysis strategy – several statistics are compared on a portion of the samples at the first stage and the most powerful statistic is then used for the remaining samples. We evaluated this procedure by mapping the quantitative trait locus of IgM with the simulated data in Genetic Analysis Workshop 15 Problem 3. The results show that the gain in power of the two-stage adaptive analysis procedure could be considerable when the initial choice of test statistic is wrong, whereas the loss is relatively small in the case that the optimal test chosen initially is correct.

## Background

Association studies currently offer an exciting approach to mapping complex quantitative trait loci (QTLs). Wallace et al. [1] recently recommended a generalized Hotelling's *T*^2 ^test for QTL linkage disequilibrium (LD) mapping, which is uniformly the best test for normally distributed data. However, if the assumption of a normal distribution is not met, *T*^2 ^may lose considerable power. When the trait distribution is unclear, some nonparametric tests may be preferred because they are only slightly less powerful than *T*^2 ^when the trait is normally distributed, but much more powerful than *T*^2 ^in some cases of non-normality. In general, it is unclear what test is the best when the trait distribution is unknown. Some investigators only report the most significant result from several statistics, but the type I error rate cannot be properly controlled when this is done. It is also not wise in this situation to use an approach such as the Bonfferoni method to control the type I error rate because the various tests are usually highly correlated and therefore the result will be overly conservative.

To achieve satisfactory power in most cases, without compromising the overall significance level, we consider adopting a two-stage adaptive analysis strategy: several statistics are compared on a portion of the samples in the first stage and the statistic that is found to be most powerful is then used for the remaining samples. Previously, two-stage strategies have been adopted in genetic association studies to reduce the cost of genotyping [2,3] or the penalty due to multiple testing when modeling gene × gene interactions [4]. Here, we apply this strategy for a different purpose: to select a powerful test for the data at hand and hence obtain good power overall. We evaluate this procedure of adaptively selecting the optimal test by mapping the IgM QTL with the simulated data of Problem 3 in Genetic Analysis Workshop 15 (GAW15).

## Methods

The procedure examines the power of various statistics using a portion of the data in an exploratory first stage and then applies this most powerful test to the rest of the data in the second stage. The statistics from the two stages are combined to make full use of the information. This approach of combining the results of the two stages is equivalent to a more general method of combining *p*-values. For the procedure of combining these *p*-values to be valid, however, we need to specify before the analysis which statistic will be used to obtain the *p*-value (*p*_1_) from the exploratory stage in the combination. The *p*-value from the second stage (*p*_2_) is calculated based on the most powerful statistic found at the first stage. Under the null hypothesis, each *p *value is, at least asymptotically, distributed uniformly on *U*(0, 1). The final decision then depends on a combining function *f*(*p*_1_, *p*_2_). The most common such function may be Fisher's combination test [5], which is defined by

*f*(*p*_1_, *p*_2_) = -2log(*p*_1_*p*_2_),

where under the null hypothesis Fisher's statistic will be distributed as a *χ*^2 ^with 4 degrees of freedom. Another example is the weighted inverse normal method,

*f*(*p*_1_, *p*_2_) = 1 - Φ[*w*_1_Φ^-1^(1 - *p*_1_) + *w*_2_Φ^-1^(1 - *p*_2_)],

where Φ is the cumulative distribution function of a standard normal distribution, 0 <*w*_*i *_< 1 and *w*_1_^2 ^+ *w*_2_^2 ^= 1. This statistic will be distributed as a standard normal distribution.

To obtain *p*_2_, we have to estimate the power of the various statistics at the exploratory first stage. Traditional power calculation methods require the trait distribution to be known, which is not the case here. A bootstrap method of using the data from the exploratory stage can be adopted to approximate the power [6,7]. The bootstrap and permutation are two often used nonparametric procedures. It is often desired to obtain "exact" *p*-values by employing a permutation procedure to generate the null distribution of the statistic that is used for a test. Here, on the other hand, we want to estimate the power of a statistic, and for this we need the distribution of the statistics under the alternative hypothesis; a permutation procedure cannot be directly applied for this purpose. Let the trait values of individuals with genotype *g *be denoted *x*_*g*_, where g = 0, 1, 2 for an additive SNP marker and g = 0, 1 for a recessive/dominant marker. For this example, we assume a dominant model for the rarer allele. We denote the sample size for each genotype *n*_*g*_. We assume the distribution of trait values for different genotypes have similar shape, but the locations of the distributions are shifted by *d*_*g*_. The hypothesis to detect association between a marker and the trait is then defined as *H*_0_:*d *= 0. The power function of the statistic *T *for d = *δ *at the significance level *α *is then given by *P*(*T*; *δ*, *α*) The method of Collings and Hamilton [6] to approximate *P*(*T*; *δ*, *α*) by a nonparametric bootstrap procedure is as follows:

1. For each genotype group *g*, a random sample of $n=\sum_{g}n_{g}$, *n*_2 _<*n*_0_, trait values is drawn with replacement. The sampled trait values are denoted *X*_*g*_^*b *^= (*x*_1*g*_^*b*^,...,*x*_*ng*_^*b*^). A simulation sample of trait values, *Y*_*g*_^*b*^, is then obtained by adding *X*_*g*_^*b *^to (**0**, ***δ***), where **0 **is a row vector of *n*_0 _elements each of which is 0 and ***δ ***is row vector of *n*_1 _+ *n*_2 _elements, each of which is *δ*. The corresponding genotype groups are set to be *G*^*b *^= (**0**, **1**).

2. Different statistics are calculated on the simulated sample values *Y*_*g*_^*b *^and *G*^*b*^, and the corresponding *p*-values (*p*_*g*_^*b*^) are recorded.

3. Steps 1 and 2 are repeated *B *times. The estimated power function of ${\hat{P}}_{g}(T;\delta ,\alpha )$ is given by $\frac{\sum I_{\left\{p_{g}^{b}<\alpha \right\}}}{B}$.

4. Finally, we estimate the power of the different statistics using the weighted average estimates of the different genotype groups, given by $\frac{\sum_{g}\left(n_{g}{\hat{P}}_{g}\right)}{n}$.

We compared non-adaptive methods and this adaptive method using the simulated data of Problem 3 in GAW15, which has 100 replicates. For an adaptive method, we considered different proportions of samples at the exploratory stage (*π*_1_), different methods of combining tests (Fisher's and the Inverse normal methods) and two statistics (Hotelling's *T*^2 ^[1] and the nonparametric Wilcoxon statistic [8]). These statistics were calculated using the R package (version 2.4.1). In each replicate, we sampled 200 independent individuals to map the IgM QTL. To examine the validity of the various tests, we randomly selected from each of the 100 replicates 10 SNPs that are not associated with IgM and therefore from these results the type I error rate is given by

$$\frac{\#\{p-value<\alpha \}}{1000}.$$

## Results

From Table 1, we can see that the two-stage analysis procedure maintains as good a type I error rate as a one-stage analysis. Table 2 shows the empirical power for the different analysis strategies. The first two rows of Tables 1 and 2 are the results from applying each of the two tests to the whole data. Because the distribution of IgM clearly deviates from a normal distribution, the loss of power of Hotelling's *T*^2 ^turns out to be severe. The two-stage analysis obtains substantial gain in power by choosing the right statistic for the second stage from "learning" at the exploratory stage. This analysis shows that using 30% of the samples at the first stage gives a good prediction of the better analytic method to use in terms of power. The results also show that the difference between the two methods of combining *p*-values is small.

**Table 1:** Type I error rate of various statistics

		*α *= 0.05		*α *= 0.01	
			
*π*_1_	Statistics	Reverse normal	Fisher	Reverse normal	Fisher
1	*T*^2^	0.032	0.032	0.010	0.010
	Wilcoxon	0.044	0.044	0.010	0.010
					
0.3	*T*^2^, *T*^2 a^	0.054	0.072	0.012	0.020
	Wilcoxon, Wilcoxon	0.050	0.054	0.010	0.014
	Adaptive 1^b^	0.050	0.054	0.010	0.014
	Adaptive 2^c^	0.056	0.048	0.014	0.01
					
0.5	*T*^2^, *T*^2 a^	0.046	0.046	0.008	0.010
	Wilcoxon, Wilcoxon	0.052	0.048	0.014	0.012
	Adaptive 1^b^	0.052	0.048	0.014	0.012
	Adaptive 2^c^	0.042	0.032	0.014	0.016

^a^The two statistics are those used at the first and second stages, respectively.

^b^The prespecified statistic used for the exploratory stage is Wilcoxon.

^c^The prespecified statistic used for the exploratory stage is *T*^2^.

**Table 2:** Power comparison of various statistics at SNP387 on chromosome 11

		*α *= 0.005		*α *= 0.001	
			
*π*_1_	Statistics	Reverse normal	Fisher	Reverse normal	Fisher
1	*T*^2^	0.35	0.35	0.09	0.09
	Wilcoxon	0.89	0.89	0.74	0.74
					
0.3	*T*^2^, *T*^2 a^	0.31	0.23	0.05	0.03
	Wilcoxon, Wilcoxon	0.86	0.81	0.61	0.60
	Adaptive 1^b^	0.86	0.81	0.61	0.60
	Adaptive 2^c^	0.76	0.69	0.51	0.43
					
0.5	*T*^2^, *T*^2 a^	0.26	0.22	0.07	0.04
	Wilcoxon, Wilcoxon	0.81	0.81	0.59	0.60
	Adaptive 1^b^	0.81	0.81	0.59	0.60
	Adaptive 2^c^	0.61	0.57	0.36	0.42

^a^The two statistics are those used at the first and second stages, respectively.

^b^The prespecified statistic used for the exploratory stage is Wilcoxon.

^c^The prespecified statistic used for the exploratory stage is *T*^2^.

## Discussion

Two-stage designs have been applied to large-scale genetic association studies to substantially reduce genotyping cost while maintaining power. In addition to the knowledge of which markers are promising, we can obtain information about the distribution of the phenotype based on the data from the exploratory stage. This knowledge is useful for the choice of a statistic to use at the second stage and can therefore lead to a considerable gain in power. In our analysis, we evaluated this idea by considering just two statistics. Hotelling's *T*^2 ^has been proved to be a powerful statistic, even with sample selection. However, the advantage of *T*^2 ^depends on the trait distribution. On the other hand, although a nonparametric statistic is not the most powerful one when normality of the trait holds, it usually works well. So it is reasonable to consider combining the *p*-value of a nonparametric statistic from the exploratory stage with the *p*-value of the most powerful statistic for the second stage.

The idea of a two-stage analysis can be further generalized in genetic association studies. Because LD patterns vary greatly, it is often unclear whether a single-marker analysis or a multiple-marker analysis or a haplotype-based analysis is most powerful for a specific data set. Further work on developing a data-driven adaptive procedure to choose the type of analysis to perform on the second stage data would be potentially useful.

## Conclusion

The adaptive two-stage procedure can lead to considerable gain in power by guiding the choice of a test based on the knowledge learned from an exploratory stage. At the same time, the type I error rate can be well controlled.

## Competing interests

The author(s) declare that they have no competing interests.
